# Supplementary material for: Identifying obstacles preventing the uptake of tunnel handling methods for laboratory mice: An international thematic survey
Source: PLoS One. 2020 Apr 14;15(4):e0231454. doi: 10.1371/journal.pone.0231454 (PMC7156035; doi:10.1371/journal.pone.0231454)
Supplement: S1 Data — (DOCX) [file pone.0231454.s001.docx]

**List of Organisations and Mailing Lists**

***Organisations & corporations:***

The National Centre for the 3Rs (NC3Rs), UK

Newcastle University Comparative Biology Centre, UK

Animal Welfare and Ethical Review Bodies Members' Knowledge Hub (AWERB), UK

The British Neuroscience Association (BNA)

The Association for the Study of Animal Behaviour (ASAB)

International Society for Neuroethology (ISN)

Center for Alternatives to Animal Testing (CAAT)

The Australian and New Zealand Council for the Care of Animals in Research and Teaching (ANZCCART)

Norway's 3R Centre and National Consensus Platform for the Replacement, Reduction and Refinement of Animal Experiments (NORECOPA)

New Zealand Ministry for Primary Industries (MPI)

Institute of Animal Technology (IAT)

Animal Welfare Research Network (AWRN)

Understanding Animal Research (UAR)

Animal Welfare Institute (AWI), USA

International Council for Laboratory Animal Science (ICLAS)

Canadian Council for Animal Care (CCAC)

Swiss 3R Competence Centre (3RCC)

Doerenkamp-Zbinden Foundation (DZF)

GSK

Novartis

***Mailing Lists:***

'Animal Welfare Science Symposium'

'behaviour-owner@yahoogroups.com';

‘BIRDSONG-L@usc.edu’

Animal Welfare Research Network

Federation of European Neuroscience Societies
