# Supplementary material for: Identifying obstacles preventing the uptake of tunnel handling methods for laboratory mice: An international thematic survey
Source: PLoS One. 2020 Apr 14;15(4):e0231454. doi: 10.1371/journal.pone.0231454 (PMC7156035; doi:10.1371/journal.pone.0231454)
Supplement: S2 Data — (PDF) [file pone.0231454.s002.pdf]

Laboratory mouse handling

Page 1

Thank you for taking the time to complete our survey on mouse handling for scientific research. The questionnaire should take no more than 15 minutes.

Page 2: About you

1. Where did you hear about this survey?

- NC3Rs
- Internal mailing list
- Professional organisation
- Colleague
- Other

1.a. If you selected Other, please specify:

2. What is your job role? ✱ *Required*

- Animal care technician
- Animal care manager
- Veterinary surgeon
- Researcher – principal investigator
- Researcher – student
- Researcher – post-doc
- Other

2.a. If you selected Other, please specify:

3. At what type of institution do you conduct your work? ✱ *Required*

- University
- Publicly Funded Research Institute
- Private Company R&D
- Other

3.a. If you selected Other, please specify:

4. In the past year, approximately how much time have you spent handling mice? (Please select one) ✱ *Required*

- Less than 1 month
- Less than 3 months
- Less than 6 months
- More than 6 months
- Every working day

5. How long have you worked with mice? (Please select one) ✱ *Required*

- Less than two years
- More than two years
- More than five years
- More than ten years

6. What country do you work in? ✱ *Required*

7. What is your age? *Optional*

- 16-25
- 26-35
- 36-45
- 46-55
- 56-65
- >65

8. What is your sex? *Optional*

- Female
- Male
- Prefer not to say

Page 3

9. Have you heard of tunnel handling methods for picking up laboratory mice? ✱
Required

- Yes
- No
- Not sure

Page 4

10. Are you aware there is evidence that tunnel handling can improve mouse welfare, and/or experimental outcomes relative to alternative handling methods? ✱ *Required*

- Yes
- No
- Not sure

Page 5

11. What methods do you use for picking up mice? Please tick all that apply.

- ☐ Tail
- ☐ Tunnel
- ☐ Cup
- ☐ Other

11.a. If you selected Other, please specify:

11.b. Why do you use tunnel handling? Please tick all the boxes that apply.

- ☐ Guidelines at place of work
- ☐ Benefits to animal welfare
- ☐ Benefits to experimental outcomes
- ☐ Not sure
- ☐ Other

11.b.i. If you selected Other, please specify:

12. If you don't use tunnel handling for picking up mice, why not? Please tick all the boxes that apply. ✱ *Required*

- ☐ Not applicable
- ☐ I had not previously heard of tunnel handling
- ☐ I use the handling methods that have always been used
- ☐ No one has suggested to do it differently
- ☐ I am concerned it will be slower
- ☐ Financial considerations; purchase of tunnels
- ☐ Financial considerations; additional staff resources
- ☐ Time required for retraining
- ☐ Access to retraining
- ☐ Possible negative influence upon experimental outcomes
- ☐ Experimental continuity
- ☐ Handling method is unimportant when mice also undergo additional invasive procedures
- ☐ Tunnel handling has not been validated for my experimental paradigm
- ☐ Not sure it's better than current method
- ☐ Not sure
- ☐ Other

12.a. If you selected Other, please specify:

12.b. Please explain your choice(s).

Page 6

Please read the main text on this page and watch the short 3-minute video that describes the implementation of tunnel handling and provides an overview of current research;

https://www.nc3rs.org.uk/how-to-pick-up-a-mouse

Page 7

13. In respect to mouse welfare, are you convinced or unconvinced by the studies conducted so far that tunnel handling is better than alternative handling methods? ✱ *Required*

Please select exactly 1 answer(s).

- ☐ 1 – not convinced
- ☐ 2 – not very convinced
- ☐ 3 – no opinion
- ☐ 4 – mildly convinced
- ☐ 5 – very convinced

13.a. Please explain your choice. ✱ *Required*

14. In respect to experimental outcomes, are you convinced or unconvinced by the studies conducted so far that tunnel handling is better than alternative handling methods? ✱ *Required*

Please select exactly 1 answer(s).

- ☐ 1 – not convinced
- ☐ 2 – not very convinced
- ☐ 3 – no opinion
- ☐ 4 – mildly convinced
- ☐ 5 – very convinced

14.a. Please explain your choice. ✱ *Required*

Page 8

15. What would be required for you to consider using tunnel handling routinely? (Please leave blank if you already use tunnel handling exclusively for picking up mice)

Your answer should be no more than 2000 characters long.

Page 9: End

Thank you for completing the survey!
