# Supplementary material for: Identifying obstacles preventing the uptake of tunnel handling methods for laboratory mice: An international thematic survey
Source: PLoS One. 2020 Apr 14;15(4):e0231454. doi: 10.1371/journal.pone.0231454 (PMC7156035; doi:10.1371/journal.pone.0231454)
Supplement: S3 Data — (DOCX) [file pone.0231454.s003.docx]

**SM3: A)** Comparison of the handling methods used by respondents between the UK (N = 119) and the other countries represented in the survey (N = 271), and **B)** a breakdown of handling methods used by Animal Care Staff and Researchers for the UK only.
